# Shoot Induction in White Eggplant (*Solanum melongena* L. Cv. Bulat Putih) using 6-Benzylaminopurine and Kinetin

**DOI:** 10.21315/tlsr2018.29.2.9

**Published:** 2018-07-06

**Authors:** Pei Ching Foo, Ze Hong Lee, Chee Keong Chin, Sreeramanan Subramaniam, Bee Lynn Chew

**Affiliations:** School of Biological Sciences, Universiti Sains Malaysia, 11800 USM Pulau Pinang, Malaysia

**Keywords:** 6-Benzylaminopurine, Kinetin, *Solanum melongena* L., Shoot Induction, 6-Benzylaminopurine, Kinetin, *Solanum melongena* L., Induksi Pucuk

## Abstract

*Solanum melongena* L. commonly known as the eggplant or brinjal comes from the family of Solanaceae, sharing the same ancestor with the tomato and potato. It is an economically important crop worldwide, being well studied for its medicinal properties, nutritional values and its role as an alternative model plant. The eggplant fruit has been previously used for treatments of various diseases such as bronchitis, asthma, arthritis and diabetes as well as its nutritive properties that are beneficial to the human diet. Plant transformation studies on the eggplant have been widely done for the production of transgenic eggplants harbouring genes that are beneficial for optimal plant growth and fruit production. Shoot induction is an essential step required for the successful regeneration of transformed plant tissues and therefore is an essential pre-requisite in *Agrobacterium-*mediated transformation. The local eggplant cv. Bulat Putih is a local cultivar of eggplant in Malaysia with white and round fruits making it a potential model plant colour pigment accumulation studies in fruit crops. The current work aims to investigate the shoot induction potential of 6-benzylaminopurine (BAP) and Kinetin from cotyledon explants of eggplant cv. Bulat Putih. Results indicated that both BAP and Kinetin were able to induce the regeneration of callus from cotyledon explants. On the other hand, Kinetin at the concentration of 2.0 mg/L successfully induced shoots at the value of 1.50 ± 0.22 shoots per explant, whereas BAP alone did not trigger any formation of shoots. This study indicated that kinetin alone is sufficient to induce shoots in eggplant cv. Bulat Putih without the presence of BAP.

## Abstract (ms)

*Solanum melongena* L. biasanya dikenali sebagai terung adalah dari famili Solanaceae, yang sama asal usulnya dengan pokok tomato dan pokok ubi kentang. Ia adalah tanaman yang penting secara ekonomi di seluruh dunia dan dikaji untuk sifat perubatannya, nilai pemakanan dan peranannya sebagai model alternatif bagi tumbuhan. Buah terung telah digunakan sejak dahulu untuk rawatan pelbagai penyakit seperti bronkitis, asma, arthritis dan kencing manis serta sifat khasiatnya yang bermanfaat kepada diet manusia. Kajian transformasi tumbuhan pada terung telah dikaji secara meluas untuk penghasilan terung transgenik yang membawa gen yang bermanfaat untuk pertumbuhan tanaman yang optimum dan pengeluaran buah yang bermutu. Penginduksian pucuk adalah langkah penting yang diperlukan untuk penjanaan semula tisu tumbuhan yang berjaya kerana ianya merupakan prasyarat penting dalam transformasi menggunakan *Agrobakterium* sebagai pengantara. Terung tempatan cv. Bulat Putih adalah kultivar terung tempatan di Malaysia dengan buah putih dan bulat menjadikannya satu model potensi untuk kajian pengumpulan pigmen warna tumbuhan untuk tanaman buah-buahan. Kajian ini bertujuan untuk menyiasat potensi penginduksian pucuk menggunakan 6-benzylaminopurine (BAP) dan Kinetin dari eksplan kotiledon terung cv. Bulat Putih. Keputusan menunjukkan bahawa kedua-dua BAP dan kinetin boleh menginduksikan regenerasi kalus dari eksplan kotiledon. Selain itu, kinetin pada kepekatan 2.0 mg/L berjaya menginduksikan pucuk pada nilai 1.50 ± 0.22 pucuk untuk setiap eksplan manakala BAP sahaja tidak dapat menginduksikan pucuk. Kajian ini menunjukkan bahawa Kinetin sahaja sudah mencukupi untuk menginduksi pucuk dalam terung cv. Bulat Putih tanpa kehadiran BAP.

## INTRODUCTION

*Solanum melongena* L. with the common name brinjal or eggplant is an economically well-known crop grown in many countries particularly at regions with tropical and temperate conditions (Collonnier *et al*. 2001). It belongs to the Solanaceae (Nightshade) family and shares the common ancestor with other well-known members in this family such as the tomato, potato, peppers and tobacco (Fukuoka *et al*. 2010). Generally, the eggplant is divided into three main categories according to the shape of their fruits. They are the egg-shaped (*S. melongena* var. *esculentum*), dwarf (*S. melongena* var. *depressum*) and long slender shaped (*S. melongena* var. *serpentium*) (Kashyap *et al*. 2003). Eggplant can grow well in regions with high rainfall as well as high temperature whereby conditions as such induces higher yield (Bhatti *et al*. 2013). The flowers of the eggplant are purplish, reddish or white in colour depending on the cultivar producing fruits either globular or long in shape, ranging from purple, white, green, brown or yellow in colour (Daunay *et al*. 2008). The eggplant is commonly known for its nutritive values as well as its medicinal properties to the human diet. Eggplant are used in cuisines worldwide especially in Asia, and also a popular ingredient in vegetarian dishes. The eggplant is packed with high soluble fiber and mineral contents as calcium, iron, potassium and phosphorus. Vitamins such vitamin C, vitamin B-6, vitamin K, folate and choline are also considerably high in the fruits making it beneficial to the human health (Bhatti *et al*. 2013). The low calorie and fat content of the eggplant fruit also contributes to weight loss and lowering risks of cardiovascular diseases (Robinson & Saranya 2013). The consumption of the eggplant fruit has also been linked to prevention of several diseases such as bronchitis, asthma, diabetes and arthritis, whereby this is mainly due to the presence of phenolic compounds such as chlorogenic acid in its fruit (Pratap *et al*. 2011; Scalzo *et al*. 2016). Previous investigations have indicated the importance of chlorogenic acid in increasing glucose tolerance in human body and subsequently reducing the risk of diabetes and obesity in human (Rodriguez & Hadley 2002; Niggeweg *et al*. 2004; Van Dijk *et al*. 2009).

Several approaches including conventional plant breeding and biotechnological methods have been employed to increase the resistance of eggplant against pest and pathogen attack. However, the introduction of resistance gene into the plant either via traditional cross breeding methods or protoplast fusion brings alongside the issues of plant sterility in progenies (Fári *et al*. 1995). Previous attempts such as *in-vitro* selection, embryo rescue, somatic hybridization and genetic engineering have greatly improved the varieties of eggplant (Swamynathan *et al*. 2010; Rattan *et al*. 2015). Intensive studies on *in-vitro* regeneration of eggplant have been successfully performed on cell suspension culture (Wang *et al*. 2013), anther culture (Salas *et al*. 2012; Rotino 2016) and *in-vitro* shoot organogenesis (Bhat *et al*. 2013; Bhatti *et al*. 2014). As reviewed by Magioli and Mansur (2005), the efficiency of *in-vitro* organogenesis of eggplant is greatly dependent on the type of explants used and the combinations of plant growth regulators supplemented.

Cytokinin and auxin are the commonly known plant growth regulators being widely used in explant regeneration as different combination ratios of these two would bring significant difference in regeneration processes. Cytokinins are commonly used in plant tissue culture to initiate cell growth and also to induce the formation of shoots whereby the are actively take part in various plant physiological processes (Davies 1995). This includes shoot multiplication (Kaviani *et al.* 2013; Bhat *et al.* 2013; Abu-Romman *et al.* 2015), tuberous root production (Deshwal & Trivedi 2011), callus induction (Borjian & Arak 2013), leaf senescence (Riefler *et al.* 2006) and shoot apical meristem activity regulators (Tucker & Laux 2007). Cytokinin such as kinetin is known to initiate cell division in the presence of auxin and is widely used together with auxins for callus formation or to induce shoots where low levels of auxin is available. Cytokinin like 6-Benzylaminopurine (BAP) or benzyl adenine on the other hand is used for growth acceleration.

Genetic transformation of crops through *Agrobacterium* mediated transformation have been utilised to introduce pest resistant genes in eggplant to increase plant survival rate and product yield (Rotino & Gleddie 1990). The establishment of *in-vitro* regeneration protocol for transformed tissues is an essential stage required of the production of transgenic plants (Billings *et al*. 1997; Franklin *et al*. 2004; Rahman *et al*. 2006). Previous transformation attempts of transformation in eggplant include introducing the genes linked to resistance to the Colorado Potato Beetle and tolerance to abiotic stresses (Arpaia *et al*. 1997; Prabhavathi *et al*. 2002; Sidhu *et al*. 2014).

Up to the current knowledge, there are no reports on the regeneration protocol for the local Malaysian white eggplant cv. Bulat Putih, particularly in *in-vitro* shoot regeneration. This white cultivar is a suitable model for transformation and genetic studies linked pigment accumulation. The current study functions as a preliminary study to assess the regeneration potential of cotyledons using cytokinins such BAP and kinetin. The current findings aim to aid future transformation studies for this cultivar particularly in genetic investigations of pigment accumulation in eggplant.

## MATERIALS AND METHODS

### Seed Surface Sterilization and *in-vitro* Germination

The seeds of round white *Solanum melongena* L. cv. Bulat Putih were obtained from Soon Huat Seeds Co. Sdn. Bhd. Penang, Malaysia. The seeds were washed with 70% ethanol for one minute, followed by rinsing with sterile distilled water for three times. Next, the seeds were washed with 50% Clorox® (commercial bleach solution) for 20 min and rinsed with sterile distilled water. Sterilised seeds were dried on sterile filter paper and placed in solid half-strength MS (Murashige & Skoog 1962) media. The sterilised seeds left to germinate in the culture room with 16 h light/8 h dark photoperiod at 24°±1°C.

### *In-vitro* Shoot Regeneration

Cotyledons obtained from 3-week-old seedlings were used as explants for shoot regeneration. The cotyledons were first excised from the seedlings and cut at both ends. The cotyledons were then cultured on solid full-strength MS medium supplemented with 6-benzylaminopurine (BAP) and Kinetin. Kinetin (0.5, 1.0, 1.5, 2.0 mg/L) and BAP (1.0, 2.0, 3.0, 4.0 mg/L) were used singly or in combinations and maintained in the culture room under 16 hours light/8 hours dark photoperiod at 24°±1°C. The explants were sub-cultured every three weeks. The percentage of callus, shoot induction and the average number of shoots induced per explant were observed and recorded after six weeks of culture.

### Statistical analysis

The experiment was performed according to the Complete Randomized Design and data were analysed using one-way analysis of variance (ANOVA) followed by comparison of means using Duncan’s multiple range test at *p* < 0.05.

## RESULTS AND DISCUSSION

**Figure 1:**
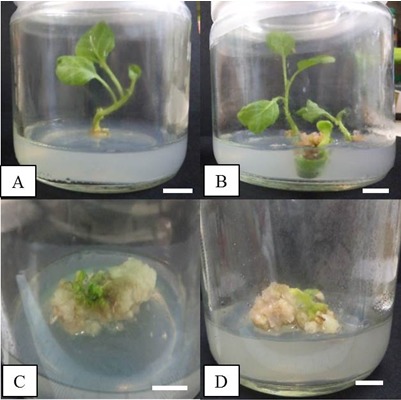
Shoot and callus formation in cotyledon explants of Solanum melongena L. cv. Bulat Putih. (A) MS medium + 1.5 mg/L kinetin. (B) MS medium + 2.0 mg/L kinetin. (C) MS medium + 3.0 mg/L BAP. (D) MS + 4.0 mg/L BAP after six weeks of culture. Scale bars represent 1 cm.

The present study describes the regeneration potential of cotyledon explants of eggplant cv. Bulat Putih. The *in-vitro* seeds germinated two weeks after surface sterilisation. In the current study, all treatments of 6-Benzylaminopurine (BAP), Kinetin and the combinations of both induced white, friable callus (Fig. 1). The calluses were able to proliferate on the same treatment medium after the first subculture. Treatments with BAP alone induced only callus but no shoot formation (Figs. 1C and 1D), indicating that media supplemented with BAP is more suitable for callus induction in the cultivar Bulat Putih. As reported by Sarker *et al*. (2006), cotyledon explants were identified to be the most suitable explant for shoot regeneration for eggplant as compared to the shoot tip, hypocotyl and roots. Previous studies on *Solanum melongena* cv. Larga Negra and Black Beauty reported the induction of callus in the treatment MS media supplemented with 0.5 mg/mL BAP + 2.0 mg/mL NAA whereby this combination favours the highest callus induction in cotyledon (90.0%) and hypocotyls (63.3%) (Zayova *et al.* 2008). On the other hand, Huda *et al.* (2007) also reported the combination of NAA and BAP initiated the formation of callus of *Solanum melongena* cv. Loda. In their research, MS media fortified with 0.05 mg/L BAP and 2.0 mg/L NAA had the highest response of callus formation from cotyledonary explants (100%). The same observation was reported by Ashrafuzzaman *et al*. (2009), whereby callus induction was the highest (95%) in MS medium supplied with 5.0 mg/mL BAP in combination with 0.1 mg/mL of NAA for hypocotyl of *Capsicum annuum* as compared to cotyledons (80%). In this study, hypocotyl explants treated with 4.0 mg/mL BAP were producing callus after 18 days of culture indicating BAP was able to induce callus for different types of explant of different Solanaceae species. Shah *et al.* (2015) also reported the formation of callus from hypocotyl explants of *Solanum lycopersicum* Mill. cv. Rio Grande in MS media supplemented with 2.0 mg/L Indole-3-acetic acid (IAA) and 2.5 mg/mL BAP (67.48 ± 0.7%).

**Figure 2:**
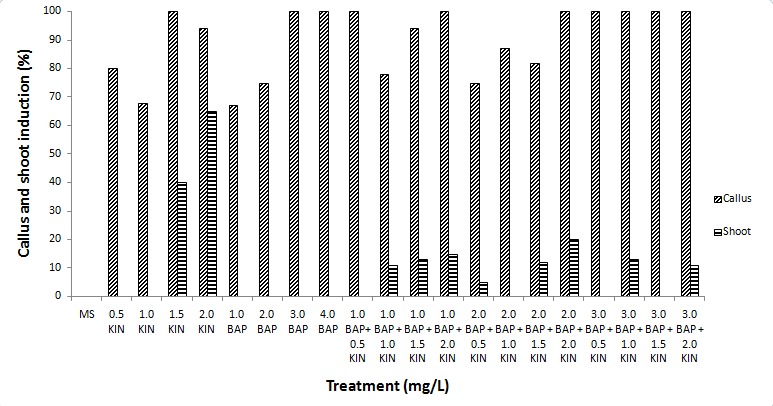
Percentage of callus and shoots induced on MS medium supplemented with different concentrations and combinations of cytokinins.

**Figure 3:**
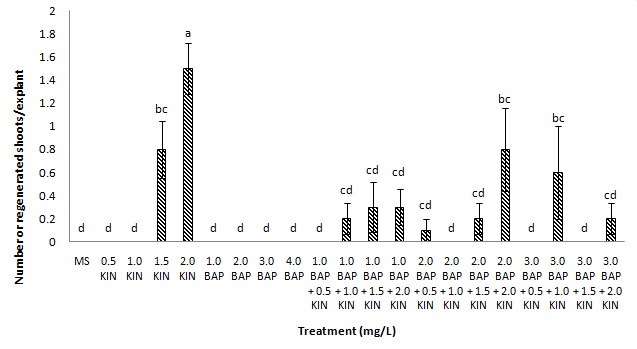
Average number of shoots regenerated on medium supplemented with different concentrations and combinations of cytokinins. Means marked by the same letters were not significantly different (Duncan Test, *p* < 0.05). Data represent mean ± standard error, *n* = 10.

Shoot formation was induced only in Kinetin supplemented media either alone or in combination with BAP. Kinetin at the concentration of 2.0 mg/L induced the highest percentage of shoot formation (65%) from the cotyledon explants with an average number of shoots of 1.50 ± 0.22 shoots per explant (Figs. 2 and 3). Shoot formation was also observed in treatments with lower concentrations of kinetin (1.5 mg/L) or in combination with BAP such as, 2.0 mg/L kinetin + 2.0 mg/L BAP and 1.0 mg/L kinetin + 3.0 mg/L BAP with average number of 0.80 ± 0.25, 0.80 ± 0.36 and 0.60 ± 0.40 shoots per explant, respectively (Fig. 3). In the current study, it was evident that the use of kinetin alone was sufficient to induce shoots from the cotyledon explants. There were no shoot and callus formation observed in full-strength MS basal medium without any plant growth regulators whereby only the formation of roots was noted. Root formation has been successfully induced on MS basal medium without plant growth regulators in other eggplant cultivars such as cv. Pusa Purple Long, cv. Black Jack and cv. Loda (Chen *et al*. 1995; Rahman *et al*. 2006; Pratap *et al*. 2011) suggesting that MS basal medium without plant growth regulators can be used for root regeneration. These results also indicated that this cultivar of *Solanum melongena* L. does not require auxins for root production.

Previous regeneration studies in *Solanum melongena* L. reported the use of kinetin in combination with other plant growth regulators to induce shoots. Sarker *et al*. (2006) used a combination of kinetin and BAP to induce a high number of shoots, ranging from 0.75 to 4.4 shoots per explant in the cultivar Singhnath and Kazla. Jamil *et al*. (2013) reported that kinetin is necessary to be included together with other plant growth regulators in inducing shoots for eggplant cultivar NS-797. They reported in their investigation the use of MS medium added with 25% of coconut milk, 1.5 mg/L of kinetin and 0.5 mg/L IAA induced shoots, whereby 70% of the total embryogenic calli produced turned green. Robinson and Saranya (2013) also reported a high number of shoot induction (5.9 ± 2.5 shoots per explant) with a percentage of 90% of shoot formation was achieved by supplementing 1.5 mg/L BAP in the culture media indicated the efficiency of BAP in shoot induction for a different cultivar. Other cytokinin such as Thidiazuron (TDZ) has also been used for shoot organogenesis of eggplant from other cultivars producing as high as 20 shoots per explant (Sharma & Rajam 1995; Magioli *et al*. 1998; 2000).

Eggplant has been used as the model plant in various fruiting plant developmental studies and the establishment of biotechnological new approaches such as embryo rescue, somatic hybridisation and *in-vitro* selection, indicated the importance of this plant species (Magioli & Mansur 2005). Up to now, various studies on eggplant has been reported mainly on the common purple variety and not much being conducted on the white cultivar. The white eggplant cultivar is an important cultivar for gene expression studies especially the expression of genes associated to the accumulation of colour pigments or phytochemicals such as carotenoids and anthocyanins. This study aids the establishment of regeneration protocol for the Malaysian local cultivar of white eggplant as a tool for future plant genetic studies on pigment accumulation in eggplant.

## CONCLUSION

MS basal medium supplemented with 2.0 mg/L of kinetin was found to induce the highest number of shoots in comparison to other treatments applied. BAP was found to be suitable for callus induction of this cultivar of eggplant instead for shoot induction. The current study is a preliminary assessment to investigate the induction shoots from the local white eggplant cultivar in Malaysia. Future studies will involve gene expression studies in this cultivar particularly colour pigment genes for quality enhancement.
